# Overall Diagnostic Efficiency of a Noninvasive Diagnostic Strategy Aimed at Early Detection of Advanced Liver Fibrosis in Metabolic Dysfunction-Associated Steatotic Liver Disease Cases

**DOI:** 10.7759/cureus.93549

**Published:** 2025-09-30

**Authors:** Kunihiro Iwata, Naoto Mori, Katsuhiko Ogasawara

**Affiliations:** 1 Department of Medical Technology, Asahikawa Medical University Hospital, Asahikawa, JPN; 2 Department Health Sciences, Hokkaido University, Sapporo, JPN

**Keywords:** advanced liver fibrosis, decision tree, efficiency, metabolic dysfunction-associated steatotic liver disease, noninvasive tests

## Abstract

Introduction

Noninvasive methods for assessing liver fibrosis are increasingly important for early detection of advanced liver fibrosis (ALF), particularly in patients with metabolic dysfunction-associated steatotic liver disease (MASLD). This study aimed to evaluate the overall diagnostic ability and efficiency (ODAE) of noninvasive diagnostic strategies incorporating imaging tests and to identify optimal test combinations for early ALF detection.

Methods

We conducted a simulation of 10,000 MASLD patients, assuming an ALF prevalence of 15%. Two diagnostic strategies were evaluated. Strategy one (two-step): patients positive on the Fibrosis-4 (Fib-4) index (cutoff ≥1.3) underwent magnetic resonance elastography (MRE), vibration-controlled transient elastography (VCTE), or shear wave elastography (SWE). Strategy two (three-step): Fib-4-positive patients underwent additional serum fibrosis marker testing - enhanced liver fibrosis score, Mac-2-binding protein glycosylation isomer, or type IV collagen 7S - followed by imaging if positive. Diagnostic performance, including net sensitivity, specificity, predictive values, and accuracy, was estimated using decision tree analysis based on published parameters.

Results

Fib-4 identified 5,742 positive cases. Strategy one achieved high accuracy (0.90-0.93); combining Fib-4 with MRE or SWE as a secondary test demonstrated good and comparable ODAE. Strategy two further improved positive predictive value and reduced false positives compared with strategy one.

Conclusions

Combining Fib-4 with MRE or SWE provides comparable and efficient diagnostic performance for early detection of ALF. Adding serum fibrosis markers as an intermediate step may further reduce false positives and enhance diagnostic accuracy.

## Introduction

Metabolic dysfunction-associated steatotic liver disease (MASLD), a condition closely linked to lifestyle-related factors, affects roughly 30% of the global adult population [1,2] and is recognized as a leading cause of chronic liver disease. In Japan, its prevalence is estimated at approximately 25% [3,4] and continues to rise. Therefore, the early and efficient detection of advanced liver fibrosis (ALF), along with timely intervention, is essential to prevent progression to cirrhosis and the emergence of related complications, particularly liver cancer.

Liver biopsy remains the most reliable method for evaluating liver fibrosis pathology, quantifying disease progression, and providing a histopathological diagnosis of the underlying condition. However, its clinical utility is limited due to several issues, including invasiveness, sampling error, procedure-related complications, and inter-observer variability among pathologists [5,6]. In light of these limitations, the importance of noninvasive methods for assessing liver fibrosis is growing. Recent guidelines now recommend a two-step diagnostic strategy (TSDS) for evaluating suspected ALF in patients with MASLD [2,7,8]. This strategy begins with noninvasive tests (NInTs) using blood-based scoring systems, such as the Fibrosis-4 (Fib-4) index or the MAFLD fibrosis score, as an initial screening tool. Patients identified as being at moderate to high risk for liver fibrosis then undergo confirmatory evaluation using either liver biopsy or elastography. A Fib-4 cutoff value of 1.3 is currently recommended for exclusion in primary screening [2]. Based on prior studies, this threshold demonstrates high sensitivity and negative predictive value (NPV), allowing ALF to be ruled out with high confidence when the Fib-4 score is below 1.3 [2]. However, because sensitivity and specificity are inherently inversely related, using the Fib-4 index alone results in a high false positive (FP) rate. To address this, as emphasized in the guidelines, a secondary diagnostic test should be performed to reduce FPs and more accurately identify high-risk patients with MASLD who require intervention. Nonetheless, the TSDS approach, which combines two diagnostic tests, not only is effective in lowering FPs compared to the Fib-4 index alone but also reduces true positive (TP) and true negative (TN) rates, potentially increasing the number of false negatives (FNs).

Among NInTs, noninvasive imaging tests (NITs) used for secondary tests, such as magnetic resonance elastography (MRE), vibration-controlled transient elastography (VCTE), and shear wave elastography (SWE), have been developed and widely adopted in clinical settings. Numerous studies have evaluated the diagnostic performance of these modalities for detecting ALF. Furthermore, hematological biomarkers such as enhanced liver fibrosis (ELF) score, type IV collagen 7S (T4C7S), and Mac-2 binding protein glycosylation isomer (M2BPGi) have recently become reimbursable under Japan’s national health insurance system. To optimize the use of the TSDS for ALF detection, it is essential to assess the overall diagnostic performance of strategies that combine these secondary tests with first-line screening methods.

The diagnostic performance of the TSDS in real-world settings has also been investigated by Japanese researchers using Japanese cohorts [6,9,10]. However, most studies assessing the ability of NITs to detect ALF have focused primarily on traditional metrics such as sensitivity, specificity, predictive values, and the area under the receiver operating characteristic curve. Although these indicators are valuable, a more nuanced understanding of diagnostic performance may be necessary. In particular, as previously noted, it is important to examine how FP and FN rates fluctuate between the primary and secondary testing phases, and how these fluctuations affect the overall diagnostic accuracy of the TSDS. Furthermore, an elevated FP rate can result in unnecessary referrals to hepatologists and may lead to overtreatment, placing an additional burden on healthcare providers. In contrast, an increase in FNs means that patients who genuinely require intervention may go undiagnosed, thereby missing the opportunity to benefit from appropriate treatment. Therefore, a clearer and more direct understanding of the relationship between diagnostic test combinations and their practical consequences is crucial for optimizing diagnostic strategies for ALF. Moreover, these comprehensive findings may have implications for other countries with similar healthcare systems, while acknowledging potential differences in population characteristics and insurance frameworks.

The aims of this study were (1) to elucidate, through simulation, the overall diagnostic ability and efficiency (ODAE) of diagnostic strategies incorporating NITs for detecting and enabling timely intervention in ALF cases among patients with MASLD, using data relevant to the Japanese population, and (2) to identify the optimal combination of serum-based and imaging-based noninvasive tests that best balances sensitivity and specificity while minimizing false results.

## Materials and methods

Study design

Our study employed a simulation methodology. The study exclusively used published literature data, without incorporating individual patient data. Consequently, ethical approval from an Institutional Review Board was not obtained. The analysis included 10,000 simulated patients aged ≥ 50 years who met the following two clinical criteria: (1) A health check revealed elevated alanine aminotransferase (ALT) (>30), and upon visiting their family doctor, it was discovered that the patient had fatty liver and one or more of the following metabolic disorders: obesity, diabetes mellitus, hypertension, and dyslipidemia; (2) There was no history of other liver diseases. The subject of the simulation was a diagnostic strategy for detecting ALF (stage F3 or higher) in outpatients who met the aforementioned clinical criteria. For the aforementioned cohort, the following clinical trajectory was postulated for simulation analysis: (1) Evaluation using the Fib-4 index was performed as the first screening step; (2) For patients with Fib-4 index ≥ 1.3, as a second examination, an imaging diagnostic examination was performed to confirm the presence or absence of ALF. The aforementioned diagnostic process was defined as the TSDS. As a baseline, the pre-test probability (PTP) of ALF was set at 15%, as previously described [11]. We assumed that the aforementioned patient group would undergo the following NITs following Fib-4 index evaluation, and the ODAE of each test was calculated using decision tree analysis [12]. The NITs assumed to be performed as secondary examinations were as follows: (1) MRE, (2) VCTE, and (3) SWE.

Literature search

A comprehensive literature review was conducted to evaluate data on the diagnostic performance of each NIT for subsequent analysis. The literature search was executed with the utmost breadth and scope possible to minimize potential bias and ensure transparency in the selection of data for analysis. The literature search was performed using the PubMed and Scopus databases to identify articles published between January 1, 2019, and September 30, 2024 (search date: October 12, 2024). The search terms were as follows: "diagnostic performance" "liver fibrosis" "metabolic dysfunction-associated steatotic liver disease"; (2) "diagnostic ability" "liver fibrosis" "metabolic dysfunction-associated steatotic liver disease"; (3) "diagnostic performance" "liver fibrosis" "nonalcoholic fatty liver disease"; and (4) "diagnostic ability" "liver fibrosis" "nonalcoholic fatty liver disease". Previous studies have demonstrated that the conventional nonalcoholic fatty liver disease (NAFLD)/nonalcoholic steatohepatitis (NASH) criteria are widely applicable to MASLD/metabolic dysfunction-associated steatohepatitis (MASH) with a diagnostic agreement rate of >95% [13-15]. Therefore, this study focused on the diagnostic performance of NITs in detecting ALF in patients with MASLD and NAFLD, which served as the primary search targets for simulation data.

Selection of articles for analysis and extraction of diagnostic ability data

From the literature search results, references that met the following criteria were selected as candidates to be employed in the analysis: (1) Studies that evaluated the diagnostic performance of NITs to detect ALF (≥ F3); (2) Studies conducted exclusively on Japanese populations; (3) Studies published within the last five years from the date of the literature search to focus on the most recent research findings; (4) Studies reporting sensitivity, specificity, and corresponding cutoff values for each NIT; (5) Studies using liver biopsy as the reference standard for diagnostic performance calculation; (6) Studies focusing solely on NAFLD and MASLD populations; (7) Studies not limited to specific patient subgroups (e.g., type 2 diabetes mellitus or obesity); and (8) Studies that examined diagnostic performance at thresholds close to those suggested in the guidelines. In serum-based NInTs, when multiple candidate sources were available, studies meeting a greater number of the following criteria were prioritized: (1) if multiple cutoff values with corresponding diagnostic ability metrics were reported, the cutoff and diagnostic ability recommended in guidelines were selected; for tests without guideline-recommended cutoffs, studies reporting high sensitivity (for rule-out purposes) were preferred; (2) multicenter study design; and (3) larger sample size. For NITs, irrespective of the above criteria, priority was given to studies that assessed the diagnostic ability of a larger number of NIT types within the same patient cohort. Finally, sensitivity and specificity data were extracted from the selected studies.

Definition of ODAE for detecting ALF

**Figure 1:**
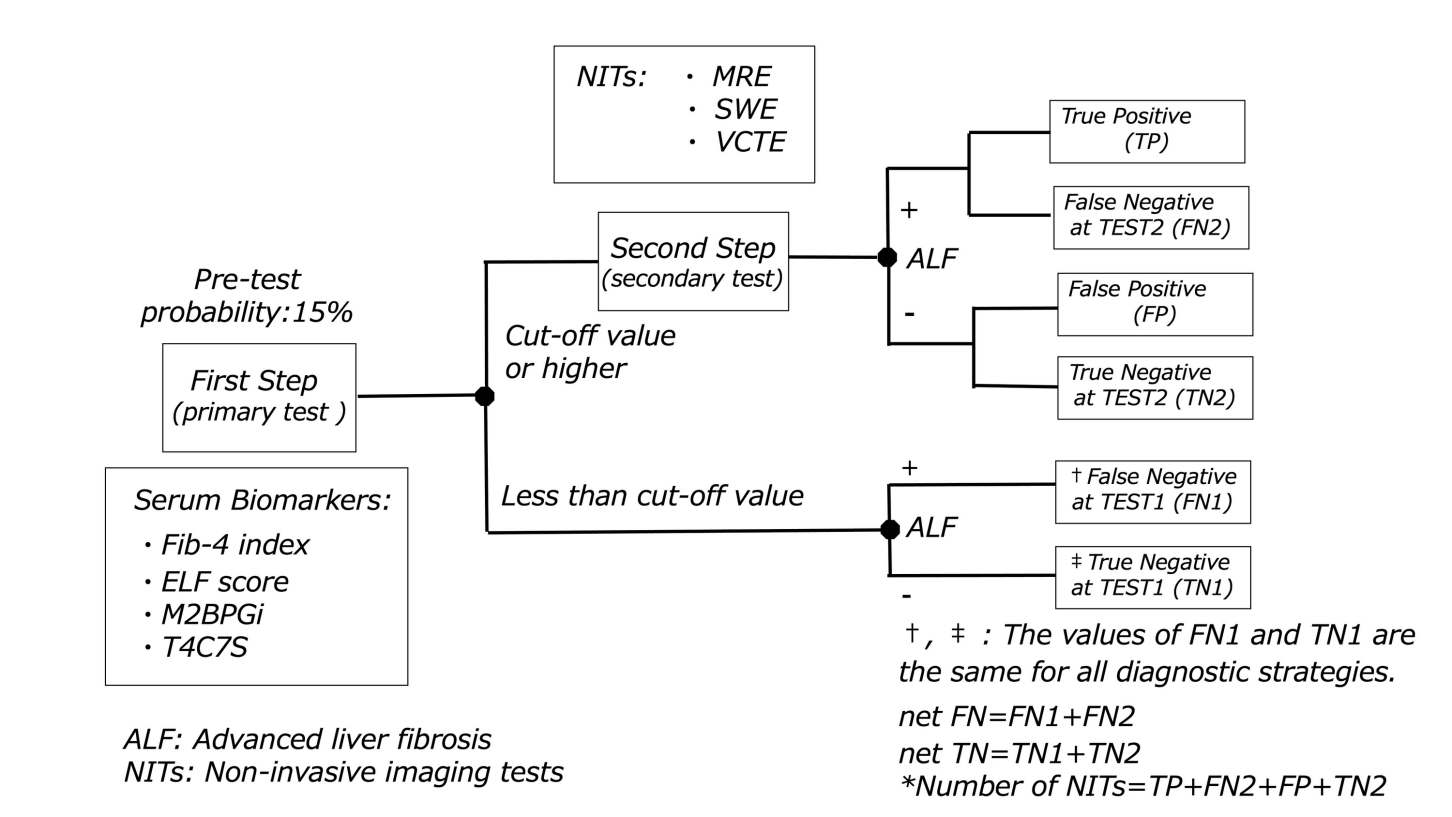
Decision tree model of two-step diagnostic strategy Fib-4 - fibrosis-4 index; ELF - enhanced liver fibrosis; T4C7S - type IV collagen 7S; M2BPGi - Mac-2 binding protein glycosylation isomer; MRE - magnetic resonance elastography; VCTE - vibration-controlled transient elastography; SWE - shear wave elastography *Number of NITs: Number of patients requiring testing by NITs based on primary test results.

In this study, we defined the indicators of ODAE for detecting ALF as follows: (1) the number of cases calculated per 10,000 patients in the TSDS (Figure 1), including true positives (TPs), false positives (FPs), false negatives in Fib-4 (FN1), true negatives in Fib-4 (TN1), false negatives in NITs (FN2), true negatives in NITs (TN2), net false negatives (FN1 + FN2), net true negatives (TN1 + TN2), and the number of patients requiring NITs based on primary or secondary test results; (2) net sensitivity and specificity (net SEN and net SP) [16]; (3) positive predictive value (PPV), defined as the post-test probability for positive results; (4) negative predictive value (NPV); (5) post-test probability for negative results (post-TP [negative results]) [17]; (6) false-positive rate (FPR) [18]; (7) false-negative rate (FNR) [18]; and (8) diagnostic accuracy (DA).

Calculation and comparison of ODAE in the TSDS

As a base case, we evaluated the ODAE of the TSDS with Fib-4 as the initial test using decision tree analysis. PTP and the sensitivity and specificity of Fib-4 and each NIT were obtained from selected studies. The final probabilities for each branch of the decision tree were calculated accordingly (Figure 1) and used to estimate the number of TPs, FPs, FNs after the first and second steps (FN1 and FN2), TNs (TN1 and TN2), and the net FN and net TN per 10,000 patients. The calculation method followed that described in a prior study [19]. Subsequently, indicators (3-8) and their 95% confidence intervals (CIs) were calculated based on the TP, FP, net FN, and net TN. For the Fib-4-only strategy, the same indicators and their CIs were derived from the TP, FP, FN1, and TN1. Finally, we compared both strategies to assess how reductions in FPs and increases in FNs influenced TP and TN outcomes. Regarding the diagnostic ability of the Fib-4 index, ODAE was evaluated for two cutoff values (1.3 and 2.0) for the elderly population [20].

ODAE of the TSDS without the Fib-4 index

**Figure 2:**
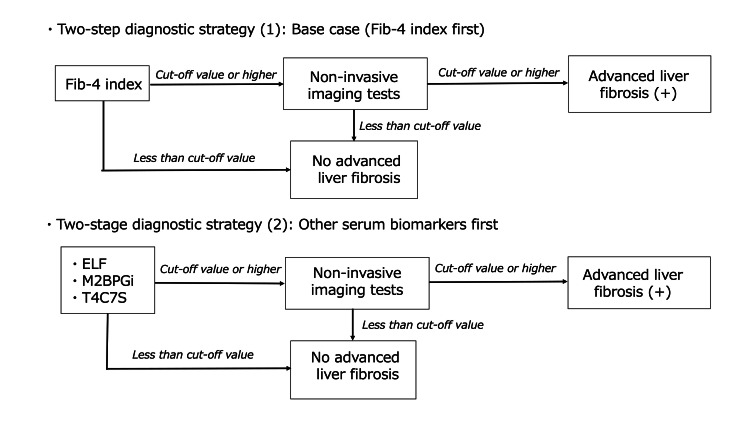
Two-step diagnostic strategy (Fib-4 index first and other serum biomarkers first) Fib-4 - fibrosis-4 index; ELF - enhanced liver fibrosis; T4C7S - type IV collagen 7S; M2BPGi - Mac-2 binding protein glycosylation isomer

The Fib-4 index is calculated from age, aspartate aminotransferase (AST), platelets, and ALT. Because the formula includes age, there is a tendency to overestimate the level of liver fibrosis in elderly individuals [21]. Therefore, a diagnostic strategy in which the Fib-4 index is not considered was assumed; alternatively, other blood tests currently covered by insurance in Japan are performed first, followed by an imaging diagnostic examination (Figure 2). In this diagnostic strategy, each indicator was also calculated and compared with the base case. Furthermore, the number of patients requiring testing with NITs based on the results of primary tests was evaluated and compared with that of the base case TSDS. The blood tests targeted in this case were as follows: (1) ELF score, (2) T4C7S, and (3) M2BPGi. Literature selection, extraction of diagnostic performance data, and PTP (15%) were applied using the same procedures as those in the base case.

Sensitivity analysis: changes in PTP

The base case PTP was set to 15%. However, this value was estimated based on a group of instances in which hepatologists performed biopsies, and this group may have a higher proportion of cases with ALF than the general population. Furthermore, to accommodate this simulation to a population with a higher PTP, an evaluation of the efficiency of different PTPs may be necessary. Therefore, a sensitivity analysis was performed to evaluate each indicator of the TSDS at different PTP levels (5%-90%). The sensitivity analysis focused solely on indicators affected by changes in the PTP. Finally, the ODAE of each TSDS was compared.

Assessment of diagnostic efficiency for a three-step diagnostic strategy

**Figure 3:**
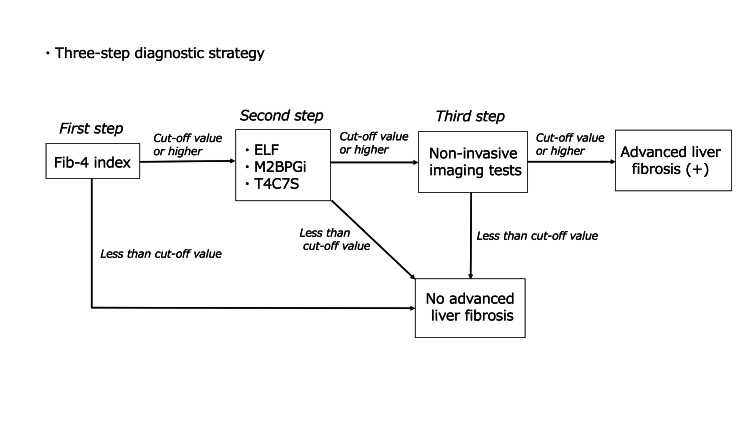
Three-step diagnostic strategy Fib-4 - fibrosis-4 index; ELF - enhanced liver fibrosis; T4C7S - type IV collagen 7S; M2BPGi - Mac-2 binding protein glycosylation isomer

It has been noted that this TSDS, which includes the Fib-4 index, offers the advantage of simplicity because of its limited steps; however, it also has the disadvantage of necessitating the referral of a substantial number of patients to centers capable of performing NITs [22]. Therefore, we assumed a three-step diagnostic strategy (Figure 3) in which patients with Fib-4 ≥ 1.3 (or 2.0 for the elderly population) underwent secondary testing using one of the three serum fibrosis markers (i.e., ELF, M2BPGi, or T4C7S), followed by NITs only for those who tested positive. Even with this diagnostic strategy, each indicator was calculated and compared with the base case. Furthermore, based on the results of primary and secondary tests, the number of patients requiring testing with NITs was evaluated and compared with that of the TSDS.

Evaluation of diagnostic efficiency and statistical analysis

Microsoft Excel for Mac 2021 (version 16.93; Microsoft, Redmond, Washington) was used for decision analysis to construct the decision tree and evaluate efficiency indicator 1. Validation of the decision tree analysis and calculation of point estimates and 95% confidence intervals for efficiency indicators (2-8) were performed using R (version 4.4.1; R Foundation for Statistical Computing, Vienna, Austria).

## Results

Literature search and articles selected for analysis

In the initial selection, we extracted five, four, one, two, two, five, and three articles on Fib-4 (12 cutoff values), ELF (13 cutoff values), M2BPGi (two cutoff values), T4C7S (three cutoff values), MRE (two cutoff values), VCTE (nine cutoff values), and SWE (three cutoff values), respectively. To select articles for the analysis, a maximum of three articles for each modality were extracted [22-30]. Among them, the article by Nouso et al. on Fib-4, which applied cutoff values of 1.3 and 2.01 for patients aged ≥60 years and satisfied criteria (1)-(3) [23], the articles by Seko et al. on ELF and M2BPGi [24] and by Kobayashi et al. on T4C7S [22], both of which also satisfied criteria (1)-(3), and the article by Imajo et al. on MRE, VCTE, and SWE, which assessed the largest number of NITs [28], met the inclusion criteria. Table 1 summarizes the patients’ characteristics.

**Table 1:** List of candidate articles and their characteristics NInTs - non-invasive tests; NITs - non-invasive imaging tests; Fib-4 - Fib-4 index; ELF - enhanced liver fibrosis; M2BPGi - Mac-2 binding protein glycosylation isomer; T4C7S - type IV collagen 7S; SWE - shear wave elastography; VCTE - vibration-controlled transient elastography; MRE - magnetic resonance elastography Articles in bold satisfied the inclusion criteria and were selected for analysis. *Results for the elderly population (≥ 60 years, only one study) Note: The results include studies that, within a single study, reported diagnostic performance at multiple cutoff values for a single non-invasive test (NInT), or reported the diagnostic performance of multiple NInTs.

Author	Year	NInTs and NITs	Study design	No. of patients	Cut-off value	Sensitivity (%)	Specificity (%)
Nouso et al. [23]	2024	Fib-4	Multicenter	1398	1.3	93.8	49
Nousoet al. [23] *	2024	Fib-4	Multicenter	606	2.01	92	41
Kobayashi et al. [22]	2022	Fib-4	Multicenter	284	1.3	91.3	43.7
Seko et al. [24]	2022	Fib-4	Multicenter	371	1.3	93.3	39
Kobayashi et al. [22]	2022	ELF	Multicenter	284	9.8	90.3	58
Seko et al. [24]	2022	ELF	Multicenter	371	9.8	91.1	50.8
Inadomi et al. [25]	2020	ELF	Multicenter	200	9.34	90.4	30.7
Seko et al. [24]	2022	M2BPGi	Multicenter	371	0.74	90.4	44.1
Seko et al. [24]	2022	M2BPGi	Multicenter	371	1.85	40	90.3
Kobayashi et al. [22]	2022	T4C7S	Multicenter	284	5.0 ng/mL	97.1	49.7
Kobayashi et al. [22]	2022	T4C7S	Multicenter	284	6.5 ng/mL	68	88.4
Shima et al. [26]	2020	T4C7S	Singlecenter	278	5.9 ng/mL	81.5	83.7
Ogino et al. [27]	2023	SWE	Singlecenter	107	1.58 m/s	77.8	78.5
Imajo et al. [28]	2022	SWE	Singlecenter	201	8.88 kPa	87	87.8
Kuroda et al. [29]	2021	SWE	Singlecenter	202	8.409 kPa	87.5	75.9
Kobayashi et al. [22]	2022	VCTE	Multicenter	284	12.0 kPa	65.5	85.6
Imajo et al. [28]	2022	VCTE	Singlecenter	201	9.7 kPa	83.6	83.3
Kuroda et al. [29]	2021	VCTE	Singlecenter	202	11.892 kPa	89.3	74.5
Imajo [28]	2022	MRE	Singlecenter	201	3.9 kPa	82.5	91.5
Tamaki et al. [30]	2019	MRE	Singlecenter	97	4.07 kPa	91.2	82.5

ODAE of the TSDS and Fib-4-only strategy

Table 2 presents the ODAE indicators for the TSDS and Fib-4-only strategy at PTP=15%. TP and FP ranged from 1161 (MRE) to 1407 (Fib-4-only) and from 368 (MRE) to 4,335 (Fib-4-only), respectively. FN2 and TN2 ranged from 183 to 246 and from 3611 to 3967, respectively. Net FN and TN ranged from 93 to 339 and from 4,165 to 8,132, respectively. Based on the Fib-4 index, 5742 patients (1407 TP + 4335 FP) required NITs. In the TSDS, SWE had the highest net SEN (81.6%) and comparable net SP (93.8%). MRE exhibited the highest PPV (75.9%) and DA (0.93), whereas its NPV (95.7%) was similar to those of others. SWE exhibited the lowest posttest probability (0.033) and FNR (0.18). MRE exhibited the lowest FPR (0.043). Compared with the Fib-4-only strategy, the TSDS significantly reduced FP and improved net SP, DA, and PPV, though with approximately 180-250 fewer TP and 190-250 more net FN, and a modest decline in net SEN. DA was significantly higher in the TSDS, with MRE and SWE yielding similar results. In the TSDS, SWE excelled in TP, net FN, net SEN, and NPV, whereas MRE excelled in FP, net TN, net SP, and PPV. The ODAE trends in the elderly population were similar (Table 3). 

**Table 2:** Comparison of ODAE between Fib-4 only strategy and various TSDS in the basic setting (PTP=15%) ODAE - overall diagnostic ability and efficiency; Fib-4 - fibrosis-4; TSDS - two-step diagnostic strategy; MRE - magnetic resonance elastography; VCTE - vibration-controlled transient elastography; SWE - shear wave elastography; CI - confidence interval;  TP - true positive; FP - false positive; FN - false negative; TN - true negative; PTP - pre-test probability † PPV - positive predictive value = post-test probability (positive result); NPV - negative predictive value ‡ Post-test probability (negative result); FPR - false positive ratio; FNR - false negative ratio * In the Fib-4 only strategy, FN1 and net FN, and TN1 and net TN are equivalent, respectively. ** In the TSDS, Fib-4-positive patients are evaluated using NITs for the presence or absence of ALF. Therefore, this sum (1407+4335) is the "number of patients who require testing by NITs" in the base case.

	Fib-4 index only	TSDS (MRE)	TSDS (VCTE)	TSDS (SWE)
Number of TP (n)	**1407	1161	1176	1224
Number of FP (n)	**4335	368	724	529
Number of FN1 (n)	-	93	93	93
Number of TN1 (n)	-	4165	4165	4165
Number of FN2 (n)	-	246	231	183
Number of TN2 (n)	-	3967	3611	3806
Number of net FN (n)	*93	339	324	276
Number of net TN (n)	*4165	8132	7776	7971
net Sensitivity (%) (95% CI)	93.8 (89.7–96.5)	77.4 (75.2–79.5)	78.4 (76.2–80.5)	81.6 (79.5–83.5)
net Specificity (%) (95% CI)	49.0 (46.1–51.9)	95.7 (95.2–96.1)	91.5 (90.9–92.1)	93.8 (93.2–94.3)
PPV (%) (95% CI) †	24.5 (23.4–25.6)	75.9 (73.7–78.1)	61.9 (59.7–64.1)	69.8 (67.6–72.0)
NPV (%) (95% CI)	97.8 (97.3–98.2)	96.0 (95.6–96.4)	96.0 (95.6–96.4)	96.7 (96.2–97.0)
Post-test probability (95% CI) ‡	0.022 (0.018–0.027)	0.040 (0.036–0.044)	0.040 (0.036–0.044)	0.033 (0.030–0.038)
FPR (95% CI)	0.51 (0.50–0.52)	0.043 (0.039–0.048)	0.085 (0.079–0.091)	0.062 (0.057–0.068)
FNR (95% CI)	0.062 (0.050–0.075)	0.23 (0.21–0.25)	0.22 (0.20–0.24)	0.18 (0.16–0.20)
Diagnostic accuracy (95% CI)	0.56 (0.55–0.57)	0.93 (0.92–0.93)	0.90 (0.89–0.90)	0.92 (0.91–0.92)

**Table 3:** Comparison of ODAE between Fib-4 only strategy and various TSDS in the elderly population (PTP=15%) ODAE - overall diagnostic ability and efficiency; Fib-4 - fibrosis-4; TSDS - two-step diagnostic strategy; MRE - magnetic resonance elastography; VCTE - vibration-controlled transient elastography; SWE - shear wave elastography; CI - confidence interval; TP - true positive; FP - false positive; FN - false negative; TN - true negative; PTP - pre-test probability † PPV - positive predictive value = post-test probability (positive result); NPV - negative predictive value ‡ Post-test probability (negative result); FPR - false positive ratio; FNR - false negative ratio * In the Fib-4 only strategy, FN1 and net FN, and TN1 and net TN are equivalent, respectively. ** In the TSDS, Fib-4-positive patients are evaluated using NITs for the presence or absence of ALF. Therefore, this sum (1380+5015) is the "number of patients who require testing by NITs" in the elderly population (≥60 years).

	Fib-4 index only	TSDS (MRE)	TSDS (VCTE)	TSDS (SWE)
Number of TP (n)	**1380	1139	1154	1201
Number of FP (n)	**5015	426	838	612
Number of FN1 (n)	-	120	120	120
Number of TN1 (n)	-	3485	3485	3485
Number of FN2 (n)	-	242	226	179
Number of TN2 (n)	-	4589	4177	4403
Number of net FN (n)	*120	362	346	299
Number of net TN (n)	*3485	8074	7662	7888
net Sensitivity (%) (95% CI)	92.0 (90.5–93.3)	75.9 (73.6–78.0)	76.9 (74.7–79.0)	80.0 (78.0–82.1)
net Specificity (%) (95% CI)	41.0 (40.0–42.1)	95.0 (94.5–95.4)	90.1 (89.5–90.8)	92.8 (92.2–93.3)
PPV (%) (95% CI) †	21.6 (20.6–22.6)	72.8 (70.5–75.0)	57.9 (55.7–60.1)	66.2 (64.0–68.4)
NPV (%) (95% CI)	96.7 (96.0–97.2)	95.7 (95.3–96.1)	95.7 (95.2–96.1)	96.3 (95.9–96.7)
Post-test probability (95% CI) ‡	0.033 (0.028–0.040)	0.043 (0.039–0.047)	0.043 (0.039–0.048)	0.037 (0.033–0.041)
FPR (95% CI)	0.59 (0.58–0.60)	0.050 (0.046–0.055)	0.099 (0.092–0.105)	0.072 (0.067–0.078)
FNR (95% CI)	0.080 (0.067–0.095)	0.24 (0.22–0.26)	0.23 (0.21–0.25)	0.20 (0.18–0.22)
Diagnostic accuracy (95% CI)	0.49 (0.48–0.50)	0.92 (0.92–0.93)	0.88 (0.88–0.89)	0.91 (0.90–0.91)

ODAE of the TSDS without the Fib-4 index

Table 4 presents the ODAE indicators for the TSDS without Fib-4 at PTP=15%. TP and FP ranged from 1119 to 1267 and from 355 to 794, respectively. FN2 and TN2 ranged from 176 to 255 and from 3484 to 4348, respectively. Net FN and TN ranged from 233 to 381 and from 7706 to 8145, respectively. The ELF+MRE strategy had the highest net SP (95.8%) and FNR (0.25) and the lowest FPR (0.042). The M2BPGi+MRE strategy exhibited the highest post-TP (negative results) (0.045). The M2BPGi+VCTE strategy had the highest FPR (0.093) and the lowest net SP (90.7%), PPV (58.8%), NPV (95.5%), and DA (0.88). The T4C7S+MRE strategy had the highest PPV (76.8%) and DA (0.93). The T4C7S+SWE strategy exhibited the highest net SEN (84.5%) and NPV (97.1%), and the lowest post-TP (negative result) (0.028) and FNR (0.16). Compared with the Fib-4-only strategy, the TSDS without Fib-4 improved the net SP, DA, and PPV and reduced the FPR, although the net SEN declined. The DA values for the ELF+MRE and T4C7S+MRE strategies (0.93) were comparable. Their FPRs (~0.04) were lower than those of others. The T4C7S+VCTE and T4C7S+SWE strategies had lower FNRs (0.19 and 0.16, respectively), and the T4C7S+SWE strategy had the highest net SEN (84.5%).

**Table 4:** Comparison of ODAE in the various TSDS without Fib-4 index (PTP=15%) ODAE - overall diagnostic ability and efficiency; ELF - enhanced liver fibrosis; M2BPGi - Mac-2 binding protein glycosylation isomer; T4C7S - type IV collagen 7S; TSDS - two-step diagnostic strategy; MRE - magnetic resonance elastography; CI - confidence interval; VCTE - vibration-controlled transient elastography; SWE - shear wave elastography; TP - true positive; FP - false positive; FN - false negative; TN - true negative; PTP - pre-test probability † PPV: Positive predictive value = post-test probability (positive result); NPV - negative predictive value ‡ Post-test probability (negative result); FPR - false positive ratio; FNR - false negative ratio * Number of patients requiring testing by NITs based on primary test results. (The difference from the base case-TSDS is shown in parentheses.)

Primary test	ELF			M2BPGi			T4C7S		
NITs	MRE	VCTE	SWE	MRE	VCTE	SWE	MRE	VCTE	SWE
Number of TP (n)	1127	1142	1189	1119	1134	1180	1202	1218	1267
Number of FP (n)	355	698	510	404	794	580	363	714	522
Number of FN1 (n)	134	134	134	144	144	144	44	44	44
Number of TN1 (n)	4318	4318	4318	3749	3749	3749	4225	4225	4225
Number of FN2 (n)	239	224	178	237	222	176	255	239	189
Number of TN2 (n)	3827	3484	3672	4348	3958	4172	3912	3561	3754
Number of net FN (n)	373	358	311	381	366	320	298	282	233
Number of net TN (n)	8145	7802	7990	8096	7706	7920	8137	7786	7978
Number of NITs (n) * (TP+FP+FN2+TN2)	5548 (-194)	5548 (-194)	5548 (-194)	6108 (-365)	6108 (-365)	6108 (-365)	5732 (-10)	5732 (-10)	5732 (-10)
net Sensitivity (%) (95% CI)	75.1 (72.9–77.3)	76.1 (73.9–78.3)	79.3 (77.1–81.3)	74.6 (72.3–76.8)	75.6 (73.3–77.8)	78.7 (76.5–80.7)	80.1 (78.0–82.1)	81.2 (79.1–83.1)	84.5 (82.5–86.3)
net Specificity (%) (95% CI)	95.8 (95.4–96.2)	91.8 (91.2–92.4)	94.0 (93.5–94.5)	95.2 (94.8–95.7)	90.7 (90.0–91.3)	93.2 (92.6–93.7)	95.7 (95.2–96.1)	91.6 (91.0–92.2)	93.9 (93.3–94.4)
PPV (%) (95% CI) †	76.0 (73.8–78.2)	62.1 (59.8–64.3)	70.0 (67.7–72.2)	73.5 (71.2–75.7)	58.8 (56.6–61.0)	67.0 (64.8–69.2)	76.8 (74.6–78.9)	63.0 (60.8–65.2)	70.8 (68.7–72.9)
NPV (%) (95% CI)	95.6 (95.2–96.0)	95.6 (95.1–96.0)	96.3 (95.8–96.7)	95.5 (95.0–95.9)	95.5 (95.0–95.9)	96.1 (95.7–96.5)	96.5 (96.1–96.9)	96.5 (96.1–96.9)	97.1 (96.8–97.5)
Post-test probability (95% CI) ‡	0.044 (0.040–0.048)	0.044 (0.040–0.049)	0.037 (0.033–0.042)	0.045 (0.041–0.050)	0.045 (0.041–0.050)	0.039 (0.035–0.043)	0.035 (0.031–0.039)	0.035 (0.031–0.039)	0.028 (0.025–0.032)
FPR (95% CI)	0.042 (0.038–0.046)	0.082 (0.076–0.088)	0.06 (0.055–0.065)	0.048 (0.043–0.052)	0.093 (0.087–0.100)	0.068 (0.063–0.074)	0.043 (0.039–0.047)	0.084 (0.078–0.090)	0.061 (0.056–0.067)
FNR (95% CI)	0.25 (0.23–0.27)	0.24 (0.22–0.26)	0.21 (0.19–0.23)	0.25 (0.23–0.28)	0.24 (0.22–0.27)	0.21 (0.19–0.23)	0.20 (0.18–0.22)	0.19 (0.17–0.21)	0.16 (0.14–0.17)
Diagnostic accuracy (95% CI)	0.93 (0.92–0.93)	0.89 (0.89–0.90)	0.92 (0.91–0.92)	0.92 (0.92–0.93)	0.88 (0.88–0.89)	0.91 (0.90–0.92)	0.93 (0.93–0.94)	0.9 (0.89–0.91)	0.92 (0.92–0.93)

Sensitivity analysis

**Figure 4:**
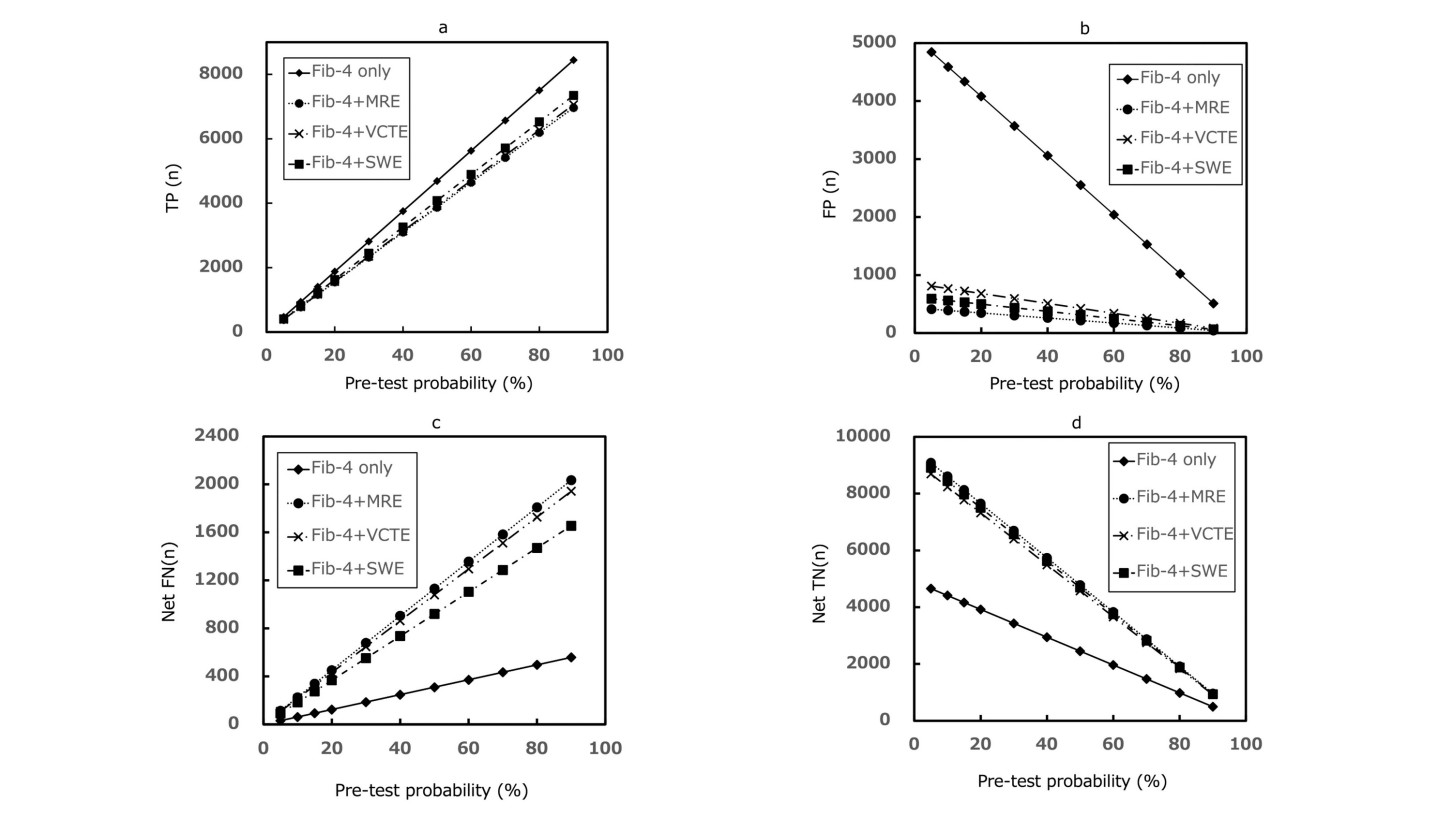
Changes in the number of diagnostic results across different pretest probabilities of ALF (TP (a), FP (b), net FN (c), net TN (d)) ALF - advanced liver fibrosis; Fib-4 - fibrosis-4 index; MRE - magnetic resonance elastography; VCTE - vibration-controlled transient elastography; SWE - shear wave elastography; TP - true positive; FP - false positive; net FN - net false negative; net TN - net true negative

**Figure 5:**
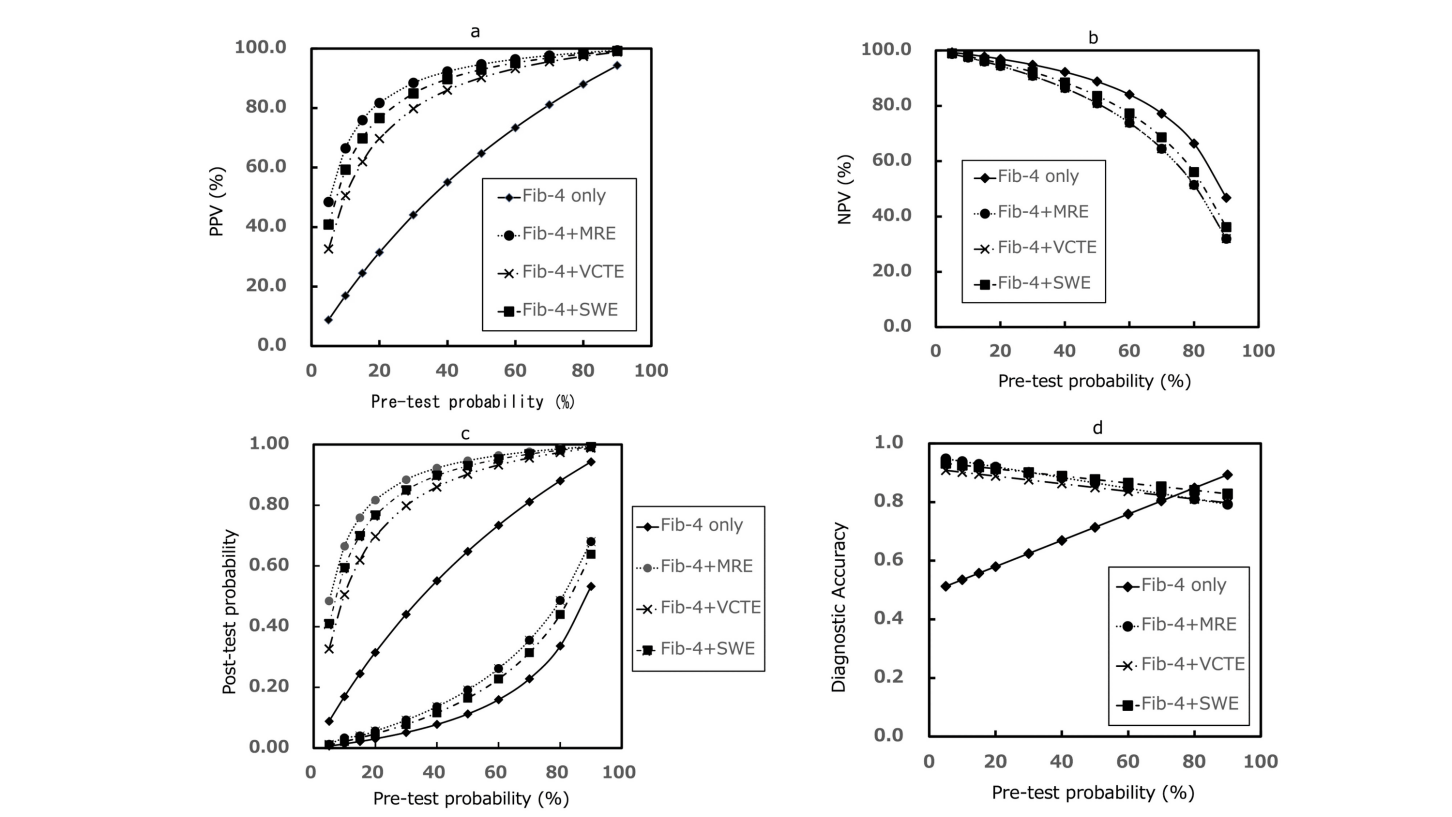
Changes in diagnostic performance metrics across different pretest probabilities of ALF (PPV (a), NPV (b), post-test probability (c), and DA (d)) Note: 1) In c, upper: post-test probability (for positive results), under: post-test probability (for negative results). 2) Post-test probability (negative results): the values for the Fib-4+MRE and Fib-4+VCTE strategies are equivalent. PPV - positive predictive value; NPV - negative predictive value; DA - diagnostic accuracy; ALF - advanced liver fibrosis; Fib-4 - fibrosis-4 index; MRE - magnetic resonance elastography; VCTE - vibration-controlled transient elastography; SWE - shear wave elastography

The sensitivity analysis showed variations in the indicators of the ODAE for each diagnostic strategy with various PTPs (Figures 4, 5). In comparing the three diagnostic strategies, the order of each indicator and other trends did not change from the baseline settings in all PTPs. Furthermore, the NPV and post-TP (negative result) of the Fib-4+MRE and Fib-4+VCTE strategies were comparable. In low-medium PTP (up to approximately 60%), compared with the Fib-4-only strategy, FP in all TSDS was reduced by approximately 1700-4000 patients; however, net FN was increased by approximately 80-900 patients. Net TN was increased by approximately 1800-4200 patients, and TP was reduced by approximately 80-900 patients. The PPV and NPV exhibited a maximum difference of 50% and 10%, respectively, and the DA exhibited a difference of approximately 0.08-0.4. The maximum difference in post-TP (negative result) was 0.1.

ODAE of the three-step diagnostic strategy

Table 5 presents the indicators for the three-step strategies. TP and FP ranged from 1049 to 1189 and from 181 to 405, respectively. Net FN and TN ranged from 311 to 451 and from 8095 to 8319, respectively. Among the strategies, the Fib-4+ELF+MRE strategy had the highest net SP (97.9%) and FNR (0.30) and the lowest FPR (0.021). The Fib-4+M2BPGi+MRE strategy exhibited the highest post-TP (negative result) (0.052) and the lowest net SEN (70.0%) and NPV (94.8%). The Fib-4+M2BPGi+VCTE strategy had the highest FPR (0.048) and the lowest net SP (95.2%), PPV (72.4%), and DA (0.92). The Fib-4+T4C7S+MRE strategy had the highest PPV (85.9%) and DA (0.94). The Fib-4+T4C7S+SWE strategy had the highest net SEN (79.3%), NPV (96.4%), and DA (0.94), and the lowest post-TP (negative result) (0.036) and FNR (0.21). Compared with the TSDS, three-step strategies improved the net SP and PPV and reduced the FP and FPR, although the TP and net SEN were lower. DA slightly improved, whereas NPV declined and FN increased, leading to a higher FNR. The Fib-4+ELF strategy required the fewest NITs (Tables 2, 5). Trends were similar between the three-step strategies and TSDS without Fib-4. Elderly population patterns were also comparable (Tables 3, 6).

**Table 5:** Comparison of ODAE in the various three-step diagnostic strategies (PTP=15%) ODAE - overall diagnostic ability and efficiency; Fib-4 - fibrosis-4 ELF - enhanced liver fibrosis; M2BPGi - Mac-2 binding protein glycosylation isomer; T4C7S - type IV collagen 7S; MRE - magnetic resonance elastography; VCTE - vibration-controlled transient elastography; SWE - shear wave elastography; TP - true positive; FP - false positive; FN - false negative; TN - true negative; NITs - non-invasive imaging tests; TSDS - two-step diagnostic strategy; SEN - sensitivity; SP - specificity; FPR - false positive ratio; FNR - false negative ratio; CI - confidence interval; PTP - pre-test probability; Fib-4 - fibrosis 4-index † PPV: Positive predictive value = post-test probability (positive result); NPV: negative predictive value; ‡ Post-test probability (negative result) * Number of patients requiring testing by NITs based on primary and secondary test results. (The difference from base case-TSDS is shown in parentheses.)

	Fib-4 index + ELF			Fib-4 index + M2BPGi			Fib-4 index + T4C7S		
NITs	MRE	VCTE	SWE	MRE	VCTE	SWE	MRE	VCTE	SWE
Number of TP (n)	1057	1072	1115	1049	1063	1107	1127	1142	1189
Number of FP (n)	181	356	260	206	405	296	185	364	266
Number of net FN (n)	443	428	385	451	437	393	373	358	311
Number of net TN (n)	8319	8144	8240	8294	8095	8204	8315	8136	8234
Number of NITs (n) *	3415 (-2327)	3415 (-2327)	3415 (-2327)	3695 (-2047)	3695 (-2047)	3695 (-2047)	3547 (-2195)	3547 (-2195)	3547 (-2195)
net SEN (%) (95% CI)	70.5 (68.1–72.8)	71.5 (69.1–73.7)	74.3 (72.0–76.5)	70.0 (67.5–72.2)	70.9 (68.5–73.2)	73.8 (71.5–76.0)	75.1 (72.9–77.3)	76.1 (73.9–78.3)	79.3 (77.1–81.3)
net SP (%) (95% CI)	97.9 (97.5–98.2)	95.8 (95.4–96.2)	96.9 (96.6–97.3)	97.6 (94.8–95.7)	95.2 (94.8–95.7)	96.5 (96.1–96.9)	97.8 (97.5–98.1)	95.7 (95.3–96.1)	96.9 (96.5–97.2)
PPV (%) (95% CI) †	85.4 (83.3–87.3)	75.1 (72.7–77.3)	81.1 (78.9–83.1)	83.6 (81.4–85.6)	72.4 (70.0–74.7)	78.9 (76.7–81.0)	85.9 (83.9–87.7)	75.8 (73.6–78.0)	81.7 (79.6–83.7)
NPV (%) (95% CI)	94.9 (94.5–95.4)	95.0 (94.5–95.5)	95.5 (95.1–96.0)	94.8 (94.4–95.3)	94.9 (94.4–95.3)	95.4 (95.0–95.9)	95.7 (95.3–96.1)	95.8 (95.3–96.2)	96.4 (95.9–96.7)
Post-test probability (95% CI) ‡	0.051 (0.046–0.055)	0.05 (0.045–0.055)	0.045 (0.040–0.049)	0.052 (0.047–0.056)	0.051 (0.047–0.056)	0.046 (0.041–0.050)	0.043 (0.039–0.047)	0.042 (0.038–0.047)	0.036 (0.033–0.041)
FPR (95% CI)	0.021 (0.018–0.025)	0.042 (0.038–0.046)	0.031 (0.027–0.034)	0.024 (0.021–0.028)	0.048 (0.043–0.052)	0.035 (0.031–0.039)	0.022 (0.019–0.025)	0.043 (0.039–0.047)	0.031 (0.028–0.035)
FNR (95% CI)	0.3 (0.27–0.32)	0.29 (0.26–0.31)	0.26 (0.23–0.28)	0.30 (0.28–0.32)	0.29 (0.27–0.32)	0.26 (0.24–0.29)	0.25 (0.23–0.27)	0.24 (0.22–0.26)	0.21 (0.19–0.23)
Diagnostic accuracy (95% CI)	0.94 (0.93–0.94)	0.92 (0.92–0.93)	0.94 (0.93–0.94)	0.93 (0.93–0.94)	0.92 (0.91–0.92)	0.93 (0.93–0.94)	0.94 (0.94–0.95)	0.93 (0.92–0.93)	0.94 (0.94–0.95)

**Table 6:** Comparison of ODAE in the various three-step diagnostic strategies (elderly population, PTP=15%) ODAE - overall diagnostic ability and efficiency; Fib-4 - fibrosis-4; ELF - enhanced liver fibrosis; M2BPGi - Mac-2 binding protein glycosylation isomer; T4C7S - type IV collagen 7S; MRE - magnetic resonance elastography; VCTE - vibration-controlled transient elastography; SWE - shear wave elastography; TP - true positive; FP - false positive; FN - false negative; TN - true negative; NITs - non-invasive imaging tests; TSDS - two-step diagnostic strategy; SEN - sensitivity; SP - specificity; FPR - false positive ratio; FNR - false negative ratio; CI - confidence interval; PTP - pre-test probability; Fib-4 - fibrosis 4-index † PPV: Positive predictive value = post-test probability (positive result); NPV: negative predictive value; ‡ Post-test probability (negative result) * Number of patients requiring testing by NITs based on primary and secondary test results. (The difference from base case-TSDS is shown in parentheses.) Definition of elderly population: ≥ 60 years

	Fib-4 index + ELF			Fib-4 index + M2BPGi			Fib-4 index + T4C7S		
NITs	MRE	VCTE	SWE	MRE	VCTE	SWE	MRE	VCTE	SWE
Number of TP (n)	1037	1051	1094	1029	1043	1085	1105	1120	1166
Number of FP (n)	210	412	301	238	468	342	214	421	308
Number of net FN (n)	463	449	406	471	457	415	395	380	334
Number of net TN (n)	8290	8088	8199	8262	8032	8158	8286	8079	8192
Number of NITs (n) *	3725 (-2670)	3725 (-2670)	3725 (-2670)	4051 (-2344)	4051 (-2344)	4051 (-2344)	3863 (-2532)	3863 (-2532)	3863 (-2532)
net SEN (%) (95% CI)	69.1 (66.7–71.5)	70.1 (67.7–72.4)	72.9 (70.6–75.1)	68.6 (66.2–70.9)	69.5 (67.1–71.9)	72.3 (70.0–74.6)	73.7 (71.4–75.9)	74.5 (72.4–76.9)	77.7 (75.5–79.8)
net SP (%) (95% CI)	97.5 (97.2–97.8)	95.2 (94.7–95.6)	96.5 (96.0–96.8)	97.2 (96.8–97.5)	94.5 (94.0–95.0)	96.0 (95.5–96.4)	97.5 (97.1–97.8)	95.0 (94.6–95.5)	96.4 (96.0–96.8)
PPV (%) (95% CI) †	83.2 (81.0–85.2)	71.8 (69.5–74.1)	78.4 (76.2–80.6)	81.2 (79.0–83.3)	69.0 (66.6–71.4)	76.0 (73.7–78.2)	83.8 (81.7–85.7)	72.7 (70.4–74.9)	79.1 (76.9–81.2)
NPV (%)	94.7 (94.2–95.2)	94.7 (94.2–95.2)	95.3 (94.8–95.7)	94.6 (94.1–95.1)	94.6 (94.1–95.1)	95.2 (94.7–95.6)	95.4 (95.0–95.9)	95.5 (95.0–95.9)	96.1 (95.6–96.5)
Post-test probability (95% CI) ‡	0.053 (0.048–0.058)	0.053 (0.048–0.058)	0.047 (0.043–0.052)	0.054 (0.049–0.059)	0.054 (0.049–0.059)	0.048 (0.044–0.053)	0.046 (0.041–0.046)	0.045 (0.041–0.050)	0.039 (0.035–0.044)
FPR (95% CI)	0.025 (0.022–0.028)	0.048 (0.044–0.053)	0.035 (0.032–0.040)	0.028 (0.025–0.032)	0.055 (0.050–0.060)	0.040 (0.036–0.045)	0.025 (0.022–0.029)	0.05 (0.045–0.054)	0.036 (0.032–0.040)
FNR (95% CI)	0.31 (0.29–0.33)	0.30 (0.28–0.32)	0.27 (0.25–0.29)	0.31 (0.29–0.34)	0.30 (0.28–0.33)	0.28 (0.25–0.30)	0.26 (0.24–0.29)	0.25 (0.23–0.28)	0.22 (0.20–0.24)
Diagnostic accuracy (95% CI)	0.93 (0.93–0.94)	0.91 (0.91–0.92)	0.93 (0.92–0.93)	0.93 (0.92–0.93)	0.91 (0.90–0.91)	0.92 (0.92–0.93)	0.94 (0.93–0.94)	0.92 (0.91–0.93)	0.94 (0.93–0.94)

## Discussion

In this study, the ODAE of diagnostic strategies was calculated using imaging tests for the early detection of ALF and identification of intervention targets in Japanese patients with MASLD, and the optimal combination of tests was determined. First, we evaluated the ODAE of the TSDS, which is recommended in Japanese guidelines. Considering the DA and the number of superior indicators, we found that the ODAE of the Fib-4+MRE and Fib-4+SWE strategies were nearly equivalent. However, when examining changes in FNs from the first to the second test, the FNR increased from 6.2% with the Fib-4-only strategy to 18% with the SWE strategy and 23% with the MRE strategy. The TSDS yielded a significant decrease in FPs and a significant increase in PPV and specificity. However, considering the absence of clear standards for acceptable increases in FNs, it is essential to fully understand these changes as trade-offs when selecting NITs.

For TSDS without Fib-4, the ELF+MRE and T4C7S+MRE or T4C7S+SWE strategies exhibited superior ODAE compared with other strategies, with similar DA values. Among the three strategies, the T4C7S+SWE strategy exhibited superiority across the greatest number of indicators, including TPs, net FNs (FNR), net SEN, NPV, and post-TP (negative). In contrast, the ODAE of the Fib-4+SWE and T4C7S+SWE strategies were nearly equivalent (Tables 2, 4). These findings suggest that the Fib-4 index, although not a dedicated fibrosis marker, can obtain roughly equivalent ODAE when used as the initial test in the TSDS. This supports current guideline recommendations.

In the three-step strategy, ODAE was superior when using the Fib-4 index as the first test, followed by either ELF as the second test and MRE as the third test (Fib-4+ELF+MRE strategy) or T4C7S as the second test with MRE or SWE as the third test (Fib-4+T4C7S+MRE and Fib-4+T4C7S+SWE strategies). The DA of these strategies was also comparable. In detailed comparisons of individual indicators among the three diagnostic strategies, the Fib-4+T4C7S+SWE strategy exhibited superiority in the greatest number of indicators (TPs, net FN (FNR), net SEN, NPV, and post-TP (negative result)). Although a slight deterioration in some indicators (decreased TP, increased FN (FNR)) was observed compared with the Fib-4+SWE strategy, the ODAE of the Fib-4+T4C7S+SWE strategy was nearly equivalent. Furthermore, compared with the Fib-4+MRE strategy, this diagnostic strategy achieved nearly identical ODAE while requiring 2,000 fewer NITs. Currently, NITs are often performed after referral to hepatologists. Our simulation results suggest that 5,742 patients with positive Fib-4 in TSDS and 5548, 6108, and 5732 patients with positive ELF, M2BPGi, and T4C7S in TSDS without Fib-4 would be referred to hepatologists for NITs or biopsy. In contrast, efficient evaluation can be achieved using a three-step diagnostic strategy in which Fib-4 and serum-based NInTs are performed initially by family physicians, with only patients suspected to be positive (3,415-3,695 patients in this study) undergoing elastography at specialized facilities. Our results demonstrated that compared with the TSDS, the three-step strategy can reduce the number of cases requiring additional NITs by approximately 2000, further reduce FPs (FPR), and provide a slight increase in TNs. Therefore, although a slight increase in testing costs (approximately 2000 JPY) is expected, using a three-step diagnostic strategy to refer only high-risk patients to specialized facilities, rather than performing advanced testing on all patients with positive Fib-4 results (≥1.3), would reduce the burden on hepatologists and enable more effective use of medical resources [22].

Previous studies have employed economic analyses, including cost-effectiveness and cost-utility analyses, to evaluate the efficiency of diagnostic strategies for ALF detection using NITs and NinTs [31,32,33]. However, interpreting efficiency indicators from these analyses requires considerable expertise. In this study, the overall sensitivity and specificity of each diagnostic strategy for ALF detection were calculated, and the ODAE of each strategy was subsequently determined using readily interpretable indicators. These parameters may provide better insight into ODAE characteristics than the sensitivity and specificity of each NInT or NIT alone. Moreover, based on PTP estimated from clinical history taking and basic laboratory tests, our results enable clinicians to prospectively assess the accuracy of ALF detection for each diagnostic strategy, including the expected number and proportion of patients in each outcome category. Our findings are also valuable for clinicians and laboratory personnel in understanding the diagnostic performance characteristics of these strategies, particularly their respective strengths and limitations.

In a previous real-world analysis, Kobayashi et al. [22] evaluated the ODAE of the TSDS for ALF detection using a combination of Fib-4 and VCTE, reporting net SEN, specificity, PPV, and NPV of 84.5%, 73.5%, 64.4%, and 89.3%, respectively. Furthermore, they evaluated the ODAE of a three-step strategy for ALF detection incorporating Fib-4, T4C7S, and VCTE, yielding net SEN, specificity, PPV, and NPV of 83.5%, 78.5%, 68.8%, and 89.3%, respectively. Our results equaled or exceeded those reported by Kobayashi et al., except for net SEN. However, these comparisons must be interpreted cautiously because our simulation-based results, derived from Bayesian calculations, were conducted under the assumption of independence among tests. In real-world practice, this assumption may not hold, as correlations between tests, clinical biases, and operational factors can lead to conditional dependence, thereby reducing the generalizability of simulation results. This methodological limitation should be taken into account when interpreting the differences between simulated and real-world diagnostic ability.

Although studies on the diagnostic potential of NITs have been published worldwide [34-36], ethnic factors [37,38] must be considered when applying or interpreting the results to the Japanese population. In contrast, evaluating the diagnostic ability and efficiency of the TSDS using data exclusively from Japan is expected to enhance the reliability of the study results when directly applied to Japanese patient populations. Furthermore, elucidating the diagnostic ability and efficiency of the TSDS using various indices and determining the appropriate combination of serum-based NInT and NITs are expected to enhance understanding of its characteristics not only among physicians but also among other medical professionals involved in testing, thereby facilitating the implementation of precise and appropriate diagnostic procedures. Moreover, if the findings of this study can support patient decision-making regarding test selection, they may also yield benefits for patients. Considering the increasing prevalence of MASLD worldwide, including in Japan, diagnostic strategies using NITs in liver fibrosis evaluation will become increasingly important in the future. 

Our study has several limitations that should be considered. First, each ODAE was derived from simulations using published data and has not been validated with real-world data. Therefore, the results of this study may not be appropriate in certain contexts. Moreover, our model relied on published parameters and did not explicitly incorporate the variability of input parameters or practical trade-offs, such as costs or accessibility, which may impose certain limitations on its applicability to real-world clinical practice. Second, although various combinations of NIT cutoff values and their corresponding diagnostic performance have been reported, our analysis used diagnostic performance values recommended in current guidelines. Because not all possible combinations were examined, superior performance from alternative combinations cannot be excluded. Third, considering the limited number of Japanese studies on NITs, the diagnostic performance (sensitivity and specificity) used in our analysis may have been over- or underestimated. To minimize potential bias and evaluate the ODAE of each strategy as objectively as possible, we prioritized studies that assessed the diagnostic performance of all three NITs in identical patient populations. However, reanalysis may be warranted as more domestic evidence accumulates. Fourth, most diagnostic performance data were derived from tertiary care facilities, primarily large hospitals. Although multicenter studies were prioritized in data selection, the calculated efficiency may remain overestimated because of potential selection bias. Finally, we did not conduct statistical significance tests when evaluating differences because our simulation included 10,000 individuals, and the resulting narrow 95% CIs for each indicator, as shown in the tables, raised concerns about overestimating statistical differences.

## Conclusions

In this study, we evaluated the ODAE of diagnostic strategies employing NITs for the detection of ALF and explored optimal combinations of NITs and NInTs. Our analysis identified several combinations that demonstrated superior ODAE: Fib-4+SWE for TSDS, T4C7S+SWE or MRE for TSDS without Fib-4, and Fib-4 index+T4C7S+MRE or SWE for the three-step strategy. These combination-based diagnostic strategies exhibited a superior balance between net sensitivity and specificity compared with the Fib-4-only strategy while reducing the FPR and achieving favorable ODAE. Our findings offer evidence-based insights to support the optimization of ALF DA and efficiency in clinical settings.
